# Numerical study of terahertz radiation from N-polar AlGaN/GaN HEMT under asymmetric boundaries

**DOI:** 10.1007/s12200-025-00148-4

**Published:** 2025-03-14

**Authors:** Runxian Xing, Hongyang Guo, Bohan Guo, Guohao Yu, Ping Zhang, Jia’an Zhou, An Yang, Yu Li, Chunfeng Hao, Huixin Yue, Zhongming Zeng, Xinping Zhang, Baoshun Zhang

**Affiliations:** 1 School of Materials Science and Engineering, Nanjing University of Science and Technology, Nanjing, 210094 China; 2 Nanofabrication Facility, Suzhou Institute of Nano-Tech and Nano-Bionics, Chinese Academy of Sciences, Suzhou, 215123 China; 3 School of Electronic Science and Engineering, University of Electronic Science and Technology of China, Chengdu, 610054 China

**Keywords:** N-polar GaN HEMT, Terahertz emission, Dyakonov–Shur instability

## Abstract

In this paper, we have studied the electrical excitation of plasma-wave in N-polar AlGaN/GaN high electron mobility transistors (HEMT) under asymmetric boundaries leads to terahertz emission. Numerical calculations are conducted through the simultaneous solution of Maxwell’s equations and the self-consistent hydrodynamic model. By employing this method, we solved the plasma-wave model in the channel of an N-polar AlGaN/GaN HEMT. We estimate that, under ideal boundary conditions and with sufficient channel mobility, these devices could generate milliwatts of power. The effects of different GaN channel layer thickness, carrier concentration, gate length and channel carrier velocity on plasma wave oscillation and terahertz radiation in N-polar AlGaN/GaN HEMT are considered. These simulation results based on Dyakonov–Shur instability provide guidance for the future design of high-radiation-power on-chip terahertz sources based on N-polar AlGaN/GaN HEMTs.

**Graphical Abstract:**

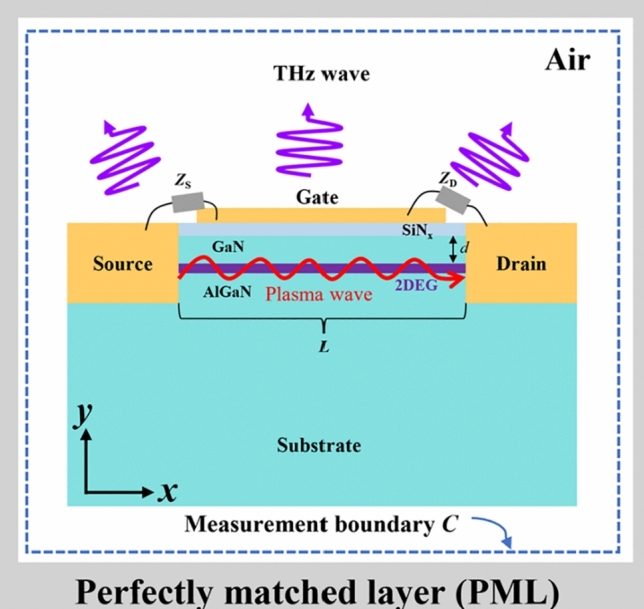

## Introduction

With the rising interest in terahertz nondestructive imaging, biological monitoring, and 6G, terahertz sources have emerged as a pivotal area of focus in cutting-edge device research [1–3]. Dyakonov and Shur, using a hydrodynamic model to analyze the dynamics of the two-dimensional (2D) electron gas in the transistor channel, predicted the potential for plasma-wave instability in short-channel high-electron-mobility transistors (HEMTs) [4]. This further indicates that, with appropriate boundary conditions and carrier concentrations in HEMT, the growth of plasma waves could extend into the terahertz frequency range. In this process, energy is converted from direct current (DC) into plasma waves, while the gain of the plasma waves must exceed the damping losses. When the energy input from the DC current reaches a dynamic equilibrium with Joule heating losses and terahertz radiation, the system attains a final stationary state.

The wide-bandgap AlGaN/GaN materials, with higher electron saturation velocity and charge density compared to AlGaAs/GaAs system, enable terahertz radiation source devices to achieve at higher operating frequencies and increased radiation power [5, 6]. To date, several studies have demonstrated the generation of terahertz radiation through the electrical excitation of plasmons in AlGaN/GaN heterojunctions on Ga-polar surfaces [7–10]. However, due to the low energy conversion efficiency, the highest terahertz radiation power achieved so far in experiments is less than 2 μW [9]. Compared to Ga-polar GaN, nitrogen-polar GaN materials have ultralow Ohmic contact resistance [11] and enhanced electron confinement [12, 13]. For N-polar GaN HEMTs, the contact resistance can reach 0.16 Ω·mm. However, by optimizing the ohmic contact conditions, the contact resistance is 0.35–0.27 Ω·mm in Ga-polar GaN HEMT. The electron sheet density in N-polar GaN HEMTs can reach 5.3 × 10^13^ cm^−2^. For Ga-polar GaN HEMTs, typical peak 2DEG densities range from 1 × 10^13^ to 2 × 10^13^ cm^−2^ in AlGaN/GaN structures under optimized conditions. Compared with Ga-polar HEMT, the calculation results of N-polar HEMT are closer to the theoretical calculated values due to its lower contact resistance. In N-polar GaN HEMTs, due to its higher electron gas concentration, it is more likely to cause plasma wave instability. Additionally, the natural back-barrier present in N-polar GaN allows for more flexible control over the gate-to-channel distance, enabling further optimization of terahertz radiation source devices based on N-polar GaN.

Several studies have investigated the use of III-V [6, 14, 15] and graphene materials [16, 17], applying asymmetric boundary conditions in the source and drain regions to induce Dyakonov–Shur (DS) instability. These studies employ multiphysics simulation platforms to self-consistently solve the hydrodynamic model and Maxwell’s equations. However, no studies have calculated the plasma wave instability in N-polar AlGaN/GaN HEMTs under DS boundary conditions. In this paper, we analyze and numerically investigate the effects of DS instability and asymmetric boundary conditions at the source and drain contacts on terahertz radiation in N-polar AlGaN/GaN HEMTs. This method simulates the hydrodynamic nonlinearity within the channel and its field coupling with the surrounding environment, thereby explaining the mechanisms linking gate length, channel layer (GaN) thickness, carrier density, and terahertz radiation. This provides guidance and a theoretical basis for designing and fabricating on-chip terahertz radiation source devices based on N-polar AlGaN/GaN HEMTs.

## Modeling methods and mechanism explanation

### Device structure

**Fig. 1:**
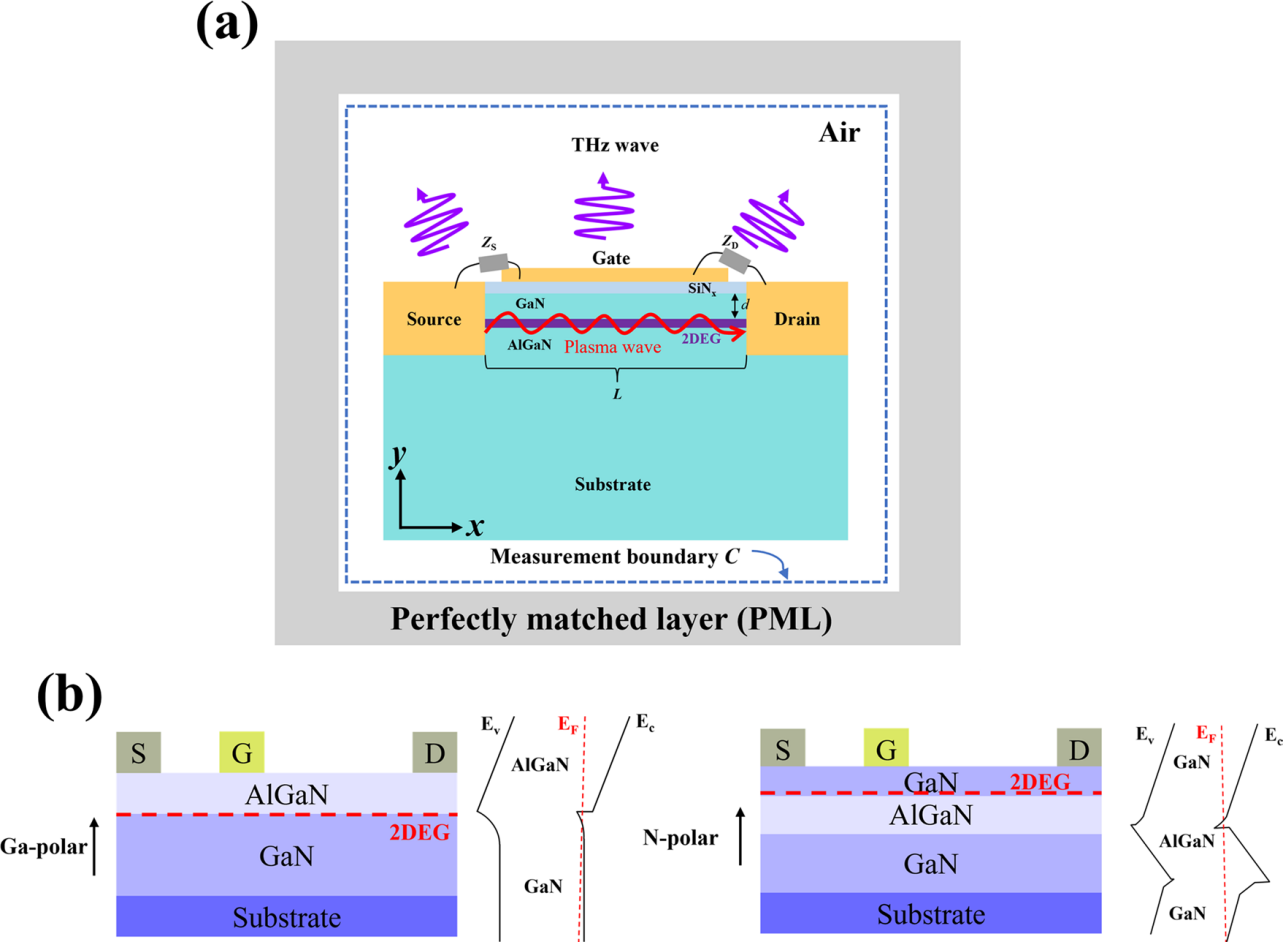
**a** Schematic of the N-polar AlGaN/GaN HEMT device structure used in numerical simulation. **b** Compare the device structures and band distribution diagrams of Ga-polar and N-polar GaN

The proposed N-polar AlGaN/GaN HEMT device is shown in Fig. 1a. The comparison of the device structures for Ga-polar and N-polar GaN is shown in Fig. 1b, in contrast to Ga-polar AlGaN/GaN HEMT devices, the GaN channel layer is positioned above the barrier layer. Due to the natural back-barrier in N-polar AlGaN/GaN HEMT devices, we approximate that the carrier concentration and mobility remain constant regardless of changes in the GaN channel layer. In fact, as the thickness of the GaN layer decreases, the carrier density also decreases, it is possible to reduce the thickness of the GaN layer while maintaining a high carrier density by adjusting the thickness of the back barrier layer [18]. This can also simplify our calculation process. It is precisely the structural differences between N-polar and Ga-polar GaN HEMTs that allow the distance between the gate and the channel in N-polar GaN to be flexibly adjustable.

In Fig. 1a, a 5 nm thick SiN layer on top of the GaN layer can reduce the surface state density of the GaN channel layer, effectively improving the stability of electron mobility and two-dimensional electron gas (2DEG) density, reducing gate leakage and preventing device performance degradation. When AlGaN and GaN are epitaxially grown on a substrate along the *c*-axis ([0001] direction), their dielectric constants are 9.5 and 9.7, respectively, assuming isotropic behavior for simplicity [19]. A thin 5 nm carrier channel layer lies between them, the channel length used in the simulation was set to 120 nm, while the back-barrier layer thickness was set to 20 nm. We also assumed that the impedance between the source and the gate is effectively zero, while the impedance between the drain and gate approaches infinity. The influence of contact resistance was neglected during the calculations. Additionally, the entire device is surrounded by an outer perfect matching layer (PML) to prevent electromagnetic waves from reflecting in the air box. At the same time, the boundary C is the boundary loop for the computing device to emit terahertz radiation to the outside, as described in Eq. (8) below.

### Principal equations

The collective oscillations of the 2DEG can exhibit fluid dynamic characteristics, allowing the electronic transport properties in the HEMT channel to be modeled using the 1D hydrodynamic model [4, 14]. In this model, suppose the 2DEG velocity along the channel is *v*(***x***, t) and carrier sheet density is *n*(***x***, t) following the continuity equations and Euler equations, as shown in Eqs. (1) and (2).

1 $$\frac{\partial n}{{\partial t}} + \frac{\partial (nv)}{{\partial x}} = 0,$$

2 $$\frac{\partial v}{{\partial t}} + v\frac{\partial v}{{\partial x}} = - \frac{e}{{m_{e}^{*} }}E_{x} - \frac{v}{\tau }.$$

Here, the electron effective mass *m*_e_* = 0.2*m*_0_ (*m*_0_ = 9.1 × 10^−31^ kg), *E*_*x*_ represents the *x*-component of the self-consistent electric field within the channel, *e* is the electron charge, and *τ* is the momentum relaxation time. The equations for current factor *j*(***x***, *t*) and surface current density *J*_*x*_(***x***, ***y***) are given by

3 $$j({\varvec{x}}, t) = n({\varvec{x}}, t)v({\varvec{x}}, t),$$

4 $$J_{x} ({\varvec{x}}, {\varvec{y}}) = - \frac{{ej({\varvec{x}}, t)}}{{t_{{2{\text{DEG}}}} }},$$

where *t*_2DEG_ is the thickness of the 2DEG layer. To calculate the electromagnetic radiation generated by plasma oscillations in the channel of N-polar GaN HEMTs, we need to perform a self-consistent solution of both the hydrodynamic equations and Maxwell’s equations. Maxwell’s equations are given as follows

5 $$\frac{{\partial E_{x} ({\varvec{x}}, {\varvec{y}})}}{\partial t} = \frac{1}{\varepsilon }\left( {\frac{{\partial H_{z} ({\varvec{x}}, {\varvec{y}})}}{\partial y} - J_{x} ({\varvec{x}}, {\varvec{y}})} \right),$$

6 $$\frac{{\partial H_{z} ({\varvec{x}}, {\varvec{y}})}}{\partial t} = \frac{1}{ - \mu }\left( {\frac{{\partial E_{y} ({\varvec{x}}, {\varvec{y}})}}{\partial x} - \frac{{\partial E_{x} ({\varvec{x}}, {\varvec{y}})}}{\partial y}} \right),$$

7 $$\frac{{\partial E_{y} ({\varvec{x}}, {\varvec{y}})}}{\partial t} = - \frac{1}{\varepsilon }\frac{{\partial H_{z} ({\varvec{x}}, {\varvec{y}})}}{\partial x}.$$

### Multiphysics simulation

The energy exchange between the two-dimensional plasmons in the channel and the terahertz radiation in free space can be achieved by simultaneously solving Maxwell’s equations and the hydrodynamic equations. We numerically solved Eqs. (1) to (7) using a custom-developed multiphysics simulation platform. Recently, this platform has been successfully employed to model DS instability in both single-channel and dual-channel FETs under externally imposed ideal boundary conditions [14, 20]. The finite-difference time domain (FDTD) method used in this study to simultaneously solve the Maxwell and hydrodynamic equations is similar to that described in Refs. [6, 15].

**Fig. 2:**
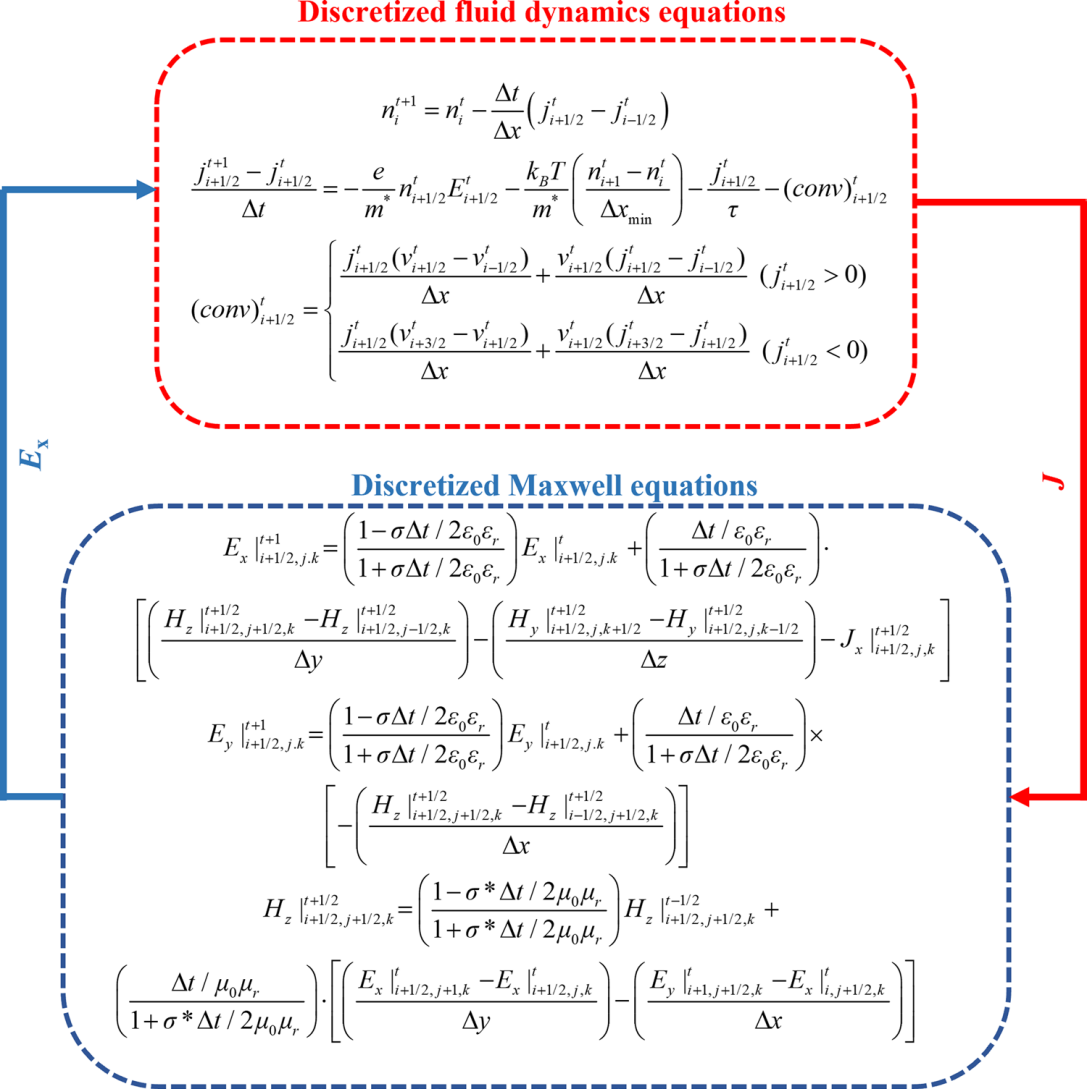
Coupling process of discretized dynamic equations and Maxwell equations

To establish asymmetric boundary conditions, where the impedance is zero (open circuit) at the source and infinite (short circuit) at the drain, we set *n*(0, *t*) = *n*_0_ and *j*(*L*, *t*) = *n*_0_*v*_0_. The DS boundary conditions similarly require *n* = *n*_0_ at the source and *j* = *n*_0_*v*_0_ at the drain. Under these ideal conditions, we assume that the impedance between the gate and the source *Z*_gs_ = 0, while the impedance between the gate and the drain *Z*_gd_ = ∞. These conditions must be incorporated into the calculations as boundary constraints throughout the simulation. In the above equation, *n*_0_ represents the equilibrium carrier concentration in the N-polar AlGaN/GaN channel, set as *n*_0_ = 2 × 10^13^ cm^−2^ [21]. Similarly to the approach in Refs. [4, 6, 22, 23], we assumed a uniform velocity for the electrons, given by *v*_0_ = −*τqV*_ds_/(*Lm*_*e*_). Here, *τ* represents the relaxation time, *q* is the electron charge, *V*_ds_ denotes the drain-source bias, the channel length *L* in the simulation was set to 120 nm, and the effective electron mass *m*_*e*_ = 0.2*m*_0_. As shown in Fig. 2, this is the time domain iterative solution process. The fluid dynamics equation is first calculated, and the initial values are assigned to the surface electron concentration *n*_0_ and drift velocity *v*_0_ in the 2DEG grid, and the ideal boundary conditions of DS are applied at both ends of the 2DEG. After the dynamics equation is solved, substitute *J* into the Maxwell equation to obtain the electric field and magnetic field components, then substitute the spatial electric field *E*_*x*_ into the dynamics equation at the next moment to drive the 2DEG layer to generate plasma wave oscillation. To enhance the stability of the hydrodynamic equation solutions, an upwind difference scheme is employed [15]. This process is repeated iteratively until the predefined computation time is reached. Additionally, we can calculate the radiated power of the terahertz source device at any given frequency by integrating the Poynting vector over the loop in the PML air domain, as shown below.

8 $$P_{{{\text{rad}}}} = \frac{1}{2}\mathop \smallint \limits_{C}^{{}} {\text{Re}} \left( {\vec{\user2{E}} \times \vec{\user2{H}}^{*} } \right) \cdot {\text{d}}\hat{\user2{l}},$$

where integration boundary *C* is noted in Fig. 1. The terms $\vec{\user2{E}}$ and $\vec{\user2{H}}$ represent the complex Fourier transforms of the electric and magnetic field vectors, respectively. And ${\text{d}}\hat{\user2{l}}$ denotes the unit vector perpendicular to boundary *C*.

**Fig. 3:**
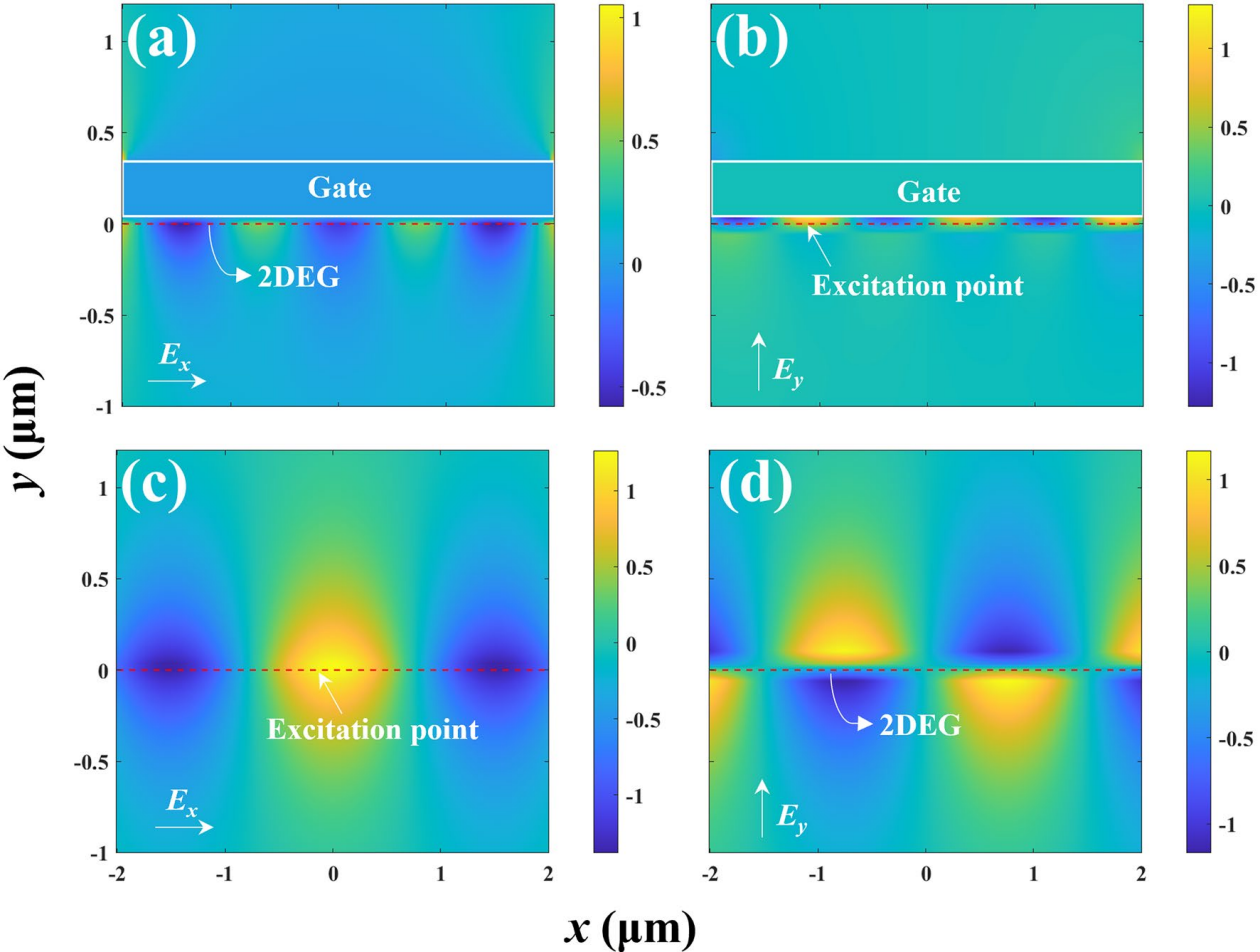
Electric field distribution in the HEMT device structure. **a** Electric field distribution in the *x*-direction of the gated region. **b** Electric field distribution in the *y*-direction of the gated region. **c** Electric field distribution in the *x*-direction of the ungated region. **d** Electric field distribution in the *y*-direction of the ungated region. It is also normalized by the peak value of the incident electromagnetic wave amplitude

To analyze the performance of this HEMT-like structure as a terahertz radiation source, we compared and plotted the electric field distribution in the *x* and *y* directions for the gated and ungated regions in Fig. 3. It is observed that plasma waves propagate away from the excitation point. Additionally, in the gated region, the electric field distribution *E*_*y*_ is significantly higher than *E*_*x*_ (due to the gate’s electric field being primarily in the vertical direction). The gate acts as a coupler, directing plasma waves in the channel to radiate as terahertz waves into free space [24]. Compared to the ungated region, the electric field distribution in the gated region indicates lower energy dissipation.

## Numerical results

### Effect of GaN channel layer thickness *d*

**Fig. 4:**
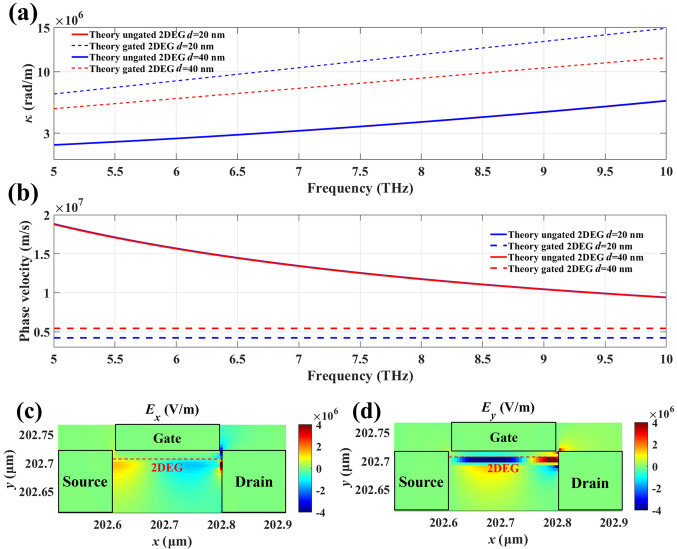
Dispersion and phase velocity model for different GaN channel layer thicknesses. **a** Dispersion diagrams for the gated and ungated regions with channel layer thicknesses of 20 and 40 nm, respectively (*n*_0_ = 2 × 10^13^ cm^−2^, m* = 0.2*m*_0_, *ε*_GaN_ = 9.7 and *ε*_AlGaN_ = 9.5). **b** Comparison of the phase velocity diagrams for the gated and ungated regions, where the channel layer thicknesses are 20 and 40 nm, respectively. **c** Electric field distribution in the *x* direction near N-polar GaN HEMTs. **d** Electric field distribution in the *y* direction near N-polar GaN HEMTs

To investigate the effect of different channel layer thicknesses on plasma wave instability, we first consider how varying the channel layer thickness affects the plasma wave vector (*k* = 2π/*λ*) and phase velocity (*v*_p_ = *ω*/*k*) as shown in Fig. 4a, b. When ignoring the scattering of the 2DEG in the channel, the dispersion relation of plasma oscillations in the ungated region from Ref. [25] is given by

9 $$k = \frac{{\omega_{{\text{p}}}^{2} m^{*} \varepsilon_{0} \overline{\varepsilon }}}{{e^{2} n_{0} }},$$

where *ω*_p_ and *k* are the angular frequency and the wave vector of plasma waves in the channel, respectively, *ε*_0_ is the dielectric constant, and *e* is the electron charge (*e* = 1.6 × 10^−19^ C). The effective dielectric constant $\overline{\varepsilon } = \frac{1}{2}\left[ {\varepsilon_\text{AlGaN} + \varepsilon_\text{GaN} \frac{{1 + \varepsilon_\text{GaN} \tanh (kd)}}{{\varepsilon_\text{GaN} + \tanh (kd)}}} \right].$ For the gated area, the gate is close enough to 2DEG (*kd* << 1), the dispersion relation obtained from [25]

10 $$k = \omega_{p} \left( {\frac{{m^{*} \varepsilon_{0} \varepsilon_\text{GaN} }}{{e^{2} n_{0} d}}} \right)^{1/2} .$$

From Eqs. (9) and (10), we can find that the plasma wave vector is independent of the gate length and the electron relaxation time. As shown in Fig. 4a, b, with the increase in GaN layer thickness, the wave vector *κ* of the 2DEG in the gated region decreases, while the wave vector *κ* in the ungated region remains unchanged. Additionally, the phase velocity of the 2DEG in the gated region increases, whereas the phase velocity in the ungated region remains constant. This indicates that the wave vector and phase velocity in the ungated region are weakly correlated with the GaN layer thickness *d*. When the wave vector remains constant, the resonant frequency increases as *d* increases.

**Fig. 5:**
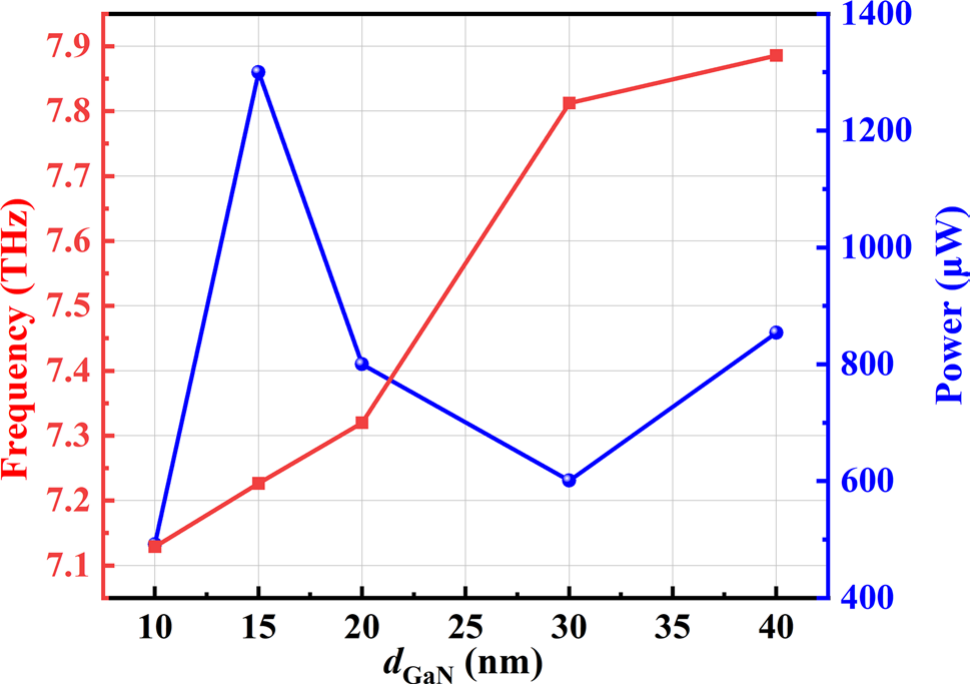
Frequency and power dependence on channel layer thickness *d* (m* = 0.2*m*_0_, *n*_0_ = 2 × 10^13^ cm^−2^, *v*_0_ = 2 × 10^5^ m/s, *L*_g_ = 100 nm and *τ* = 1 × 10^−12^ s)

The terahertz radiation power at different GaN layer thicknesses *d* can be obtained using Eq. (8), as illustrated in Fig. 5. As shown in Fig. 5, the resonant frequency increases with the increase in channel layer thickness. This is because varying the GaN layer thickness alters the wave vector in the gated region. As shown in Fig. 4a, when the wave vector remains constant, the resonant frequency increases with the increase of the GaN layer thickness. As shown in Fig. 5, when the GaN layer thickness increases from 20 to 40 nm, the resonance frequency only increases by 0.6 THz, indicating a relatively insignificant change. From Fig. 4a, b, it can be observed that as the thickness of the GaN layer increases from 20 to 40 nm, the resonance frequency in the gated region increases by 2 THz for the same wave vector, while the resonance frequency in the ungated region remains unchanged. This is because the coupling between the plasmon waves in a single gated region and the terahertz wave is relatively weak.

The electric field distributions along the *x* and *y* directions in Fig. 4c, d reveal that, due to the shielding effect of the gate, the electric field is predominantly concentrated in the ungated regions at the edges of the gate. The gate, acting as a coupler, cannot directly emit terahertz waves. Terahertz radiation is generated only when the plasmons in the gated region strongly interact with the ungated region, and the resulting energy is radiated from the ungated area [25]. In other words, the terahertz radiation generated by the device is predominantly governed by plasmons in the ungated regions. Additionally, as seen in Fig. 5, our calculations indicate that with a gate length of 100 nm, the maximum radiation power of 1300 μW. As the GaN layer thickness increases, the terahertz radiation power initially rises due to the increase in the wave vector. However, with further increases in GaN layer thickness, the coupling between the gate and the plasma waves in the channel weakens, resulting in a decrease in radiation power.

### Effect of gate length *L*_g_

**Fig. 6:**
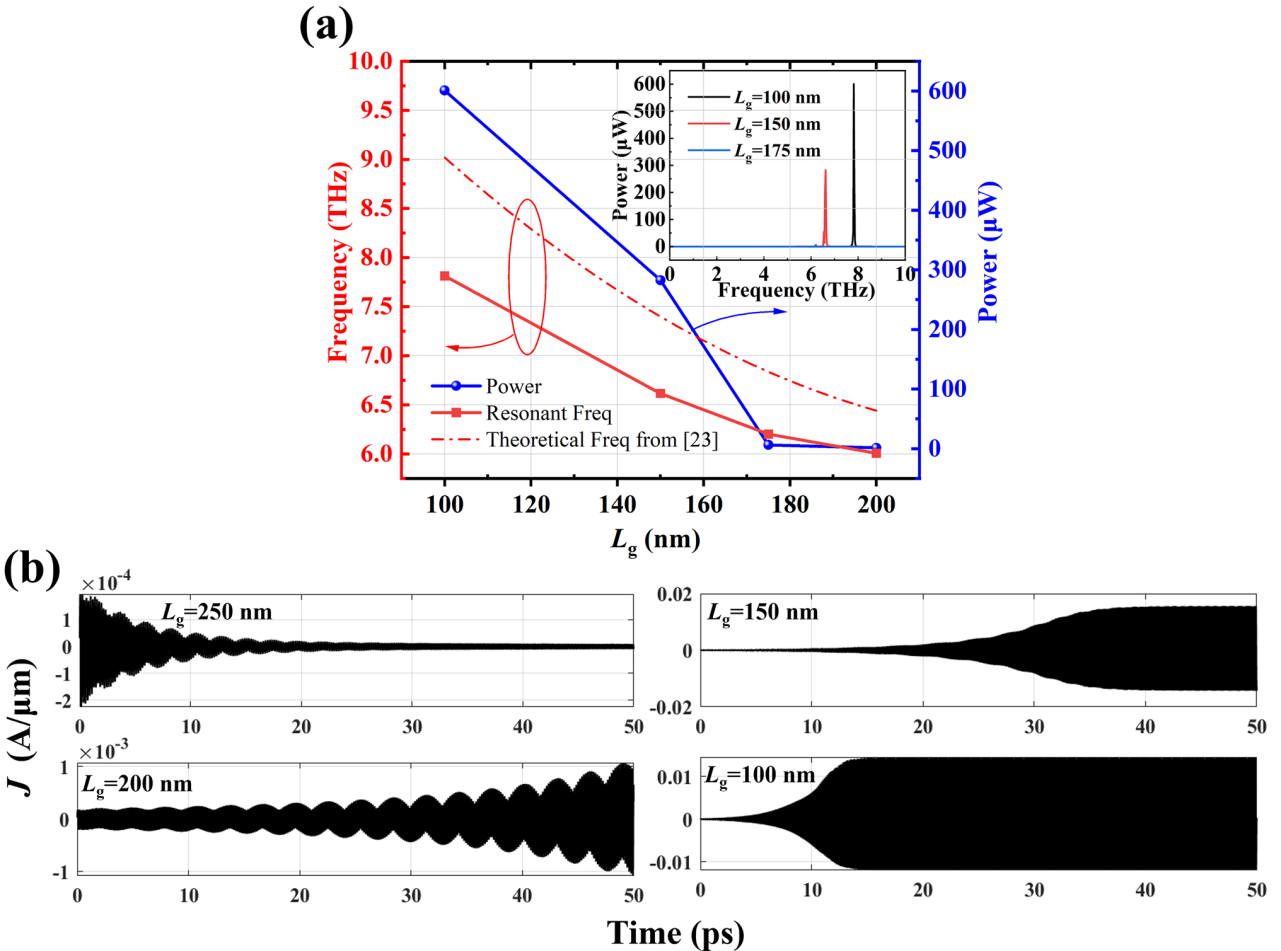
**a** Frequency and power dependence on gate length, *L*_g_. The theoretical calculation model is described in Ref. [23]. **b** Current density versus time for different gate lengths (m* = 0.2*m*_0_, *n*_0_ = 2 × 10^13^ cm^−2^, *v*_0_ = 2 × 10^5^ m/s, *d*_GaN_ = 30 nm and *τ* = 1 × 10^−12^ s)

In Fig. 6a, we compare the resonant frequency and radiation power for different gate lengths.

When the gate length is 100 nm, through our multiphysics simulation platform calculations show that the maximum radiation power of the N-polar AlGaN/GaN structure can reach up to 600 µW with *n*_0_ = 2 × 10^13^ cm^−2^, *v*_0_ = 2 × 10^5^ m/s, *d*_GaN_ = 30 nm and *τ* = 1 × 10^−12^ s. We found that as the gate length increases, the total radiation power gradually decreases. This is because, according to plasma instability theory, shorter gate lengths result in a higher amplification factor and lower power dissipation for plasma waves [7]. Theoretically, the frequencies of ungated plasmons of various orders, governed by DS boundary conditions, are determined by [23]

11 $$\omega = \sqrt {\frac{{e^{2} n_{0} }}{{2\varepsilon m^{*} L_\text{g} }}\left( {\frac{\pi }{2} + n\pi } \right)} ,\,n = 0, \, 1, \, 2, \, 3 \ldots$$

Figure 6a also shows that as the gate length increases, the resonant frequency decreases, which is consistent with the trend predicted by theoretical calculations in Ref. [23] (*n* = 1 in Eq. (10)). However, we observe that the plasma resonance frequency obtained from calculations is lower than that predicted by the theoretical model. This discrepancy arises because plasma wave resonance may occur in the ungated regions at the edges of the gate, which can be larger than the expected quarter-wavelength [23]. The inset in Fig. 6a compares the power spectra at different gate lengths. It can be seen that the gate length has a significant impact on the resonant frequency and radiation power. As shown in Fig. 6b, the current variation with time for different gate lengths indicates that shorter gate lengths lead to higher radiation power due to reduced energy dissipation and increased current gain. When the current gain and energy dissipation reach dynamic equilibrium, the oscillation of the current density reaches saturation and stabilizes. The shorter of gate length, the shorter propagation distance, and the less time is required to reach steady-state. If the gate length is further increased (*L*_g_ = 250 nm), energy dissipation exceeds current gain, and current oscillations no longer occur. In other words, when the gate length is 250 nm, the condition 2*v*_0_/*L*_g_ > 1/*τ* is no longer satisfied, and plasma wave oscillations cease to occur.

### Effect of channel carrier sheet density ${n}_{0}$ and channel carrier velocity ${v}_{0}$

**Fig. 7:**
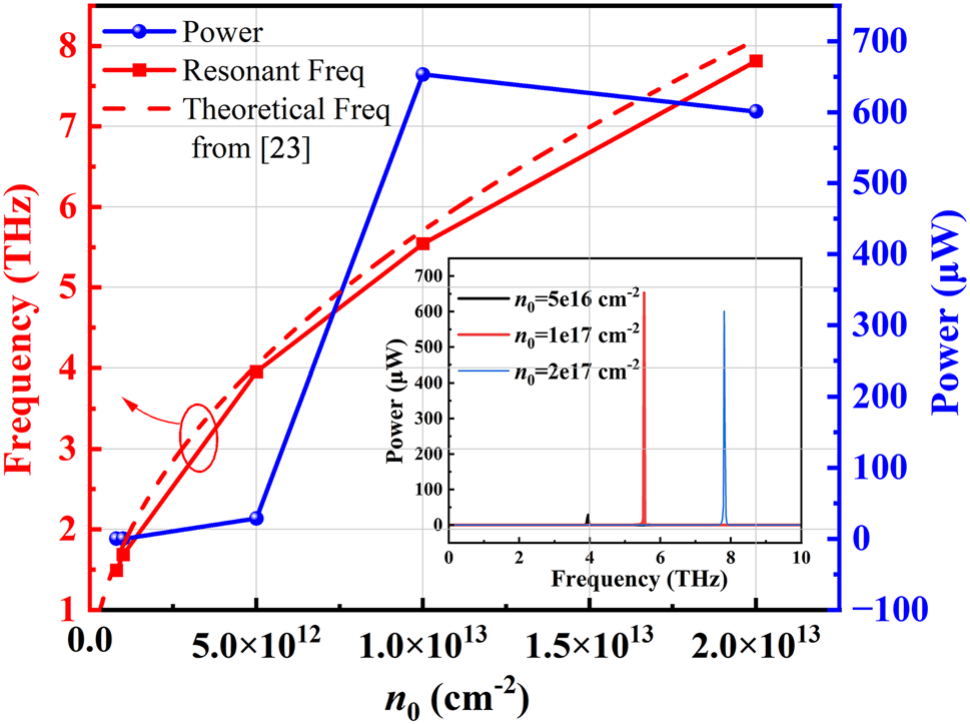
Frequency and power dependence on channel carrier sheet density, ${n}_{0}$. The theoretical calculation model is described in Ref. [23] (m* = 0.2*m*_0_, *L*_g_ = 100 nm, *v*_0_ = 2 × 10^5^ m/s, *d*_GaN_ = 30 nm and *τ* = 1 × 10^−12^ s)

Figure 7 shows the resonance frequencies and corresponding emission power with changing channel carrier sheet density *n*_0_. We observe that in N-polar GaN HEMTs, the resonant frequency increases as the carrier density in the channel rises. This simulation result is consistent with previous simulations based on Ga-polar GaN HEMTs [6]. It can be observed that the surface electron concentration significantly affects the resonance frequency. It can also be seen that the resonant frequency calculated using our simulation platform is very consistent with the theoretical formula results in Ref. [23]. This is because we assume that the metal contacts in the HEMT are ideal at terahertz frequencies. In addition, as the carrier density increases, the radiation power gradually rises until saturation and then slightly decreases. This is because at lower electron gas concentrations, as the concentration increases, the coupling effect between the plasma wave and the electric field is enhanced, leading to an increase in output power. However, as the electron gas concentration continues to increase, coulomb scattering between carriers and interactions with the lattice become more pronounced, resulting in a decrease in electron mobility. Consequently, the intensity of coherent terahertz radiation generated by DS instability is reduced. In Ga-polar GaN HEMTs, it has also been observed that as the electric field in the channel increases, the terahertz radiation power gradually increases until saturation and then decreases [8]. Terahertz radiation power in the range of several hundred microwatts can be expected from N-polar GaN HEMTs when *L*_g_ = 100 nm, *v*_0_ = 2 × 10^5^ m/s, *d*_GaN_ = 30 nm and *τ* = 1 × 10^−12^ s. Moreover, the inset of Fig. 7 shows the emission spectra for different channel carrier densities as previously described.

**Fig. 8:**
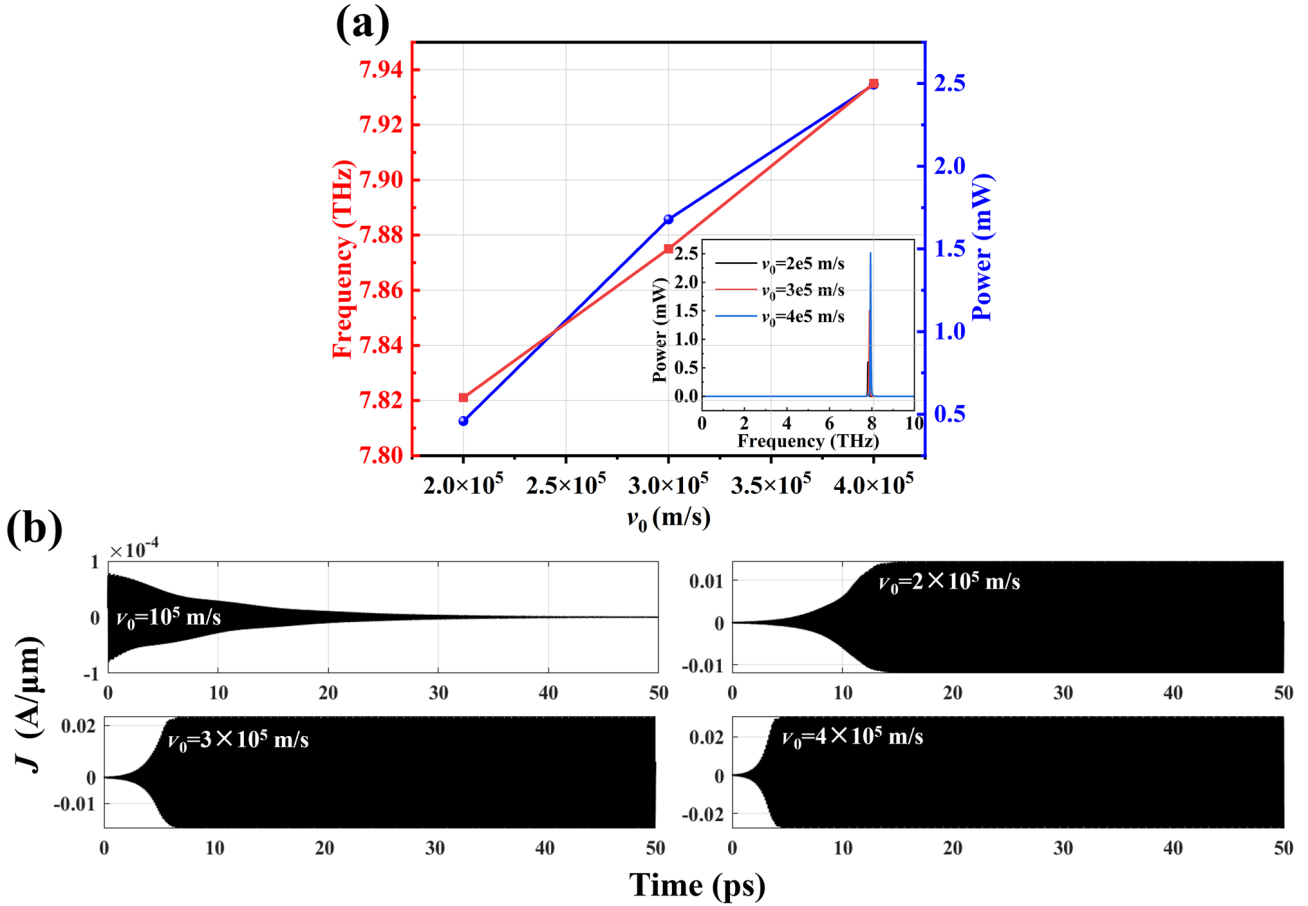
**a** Frequency and power dependence on channel carrier velocity, *v*_0_. **b** Relationship between current density and time at different electron drift velocities (m* = 0.2*m*_0_, *n*_0_ = 2 × 10^13^ cm^−2^, *L*_g_ = 100 nm, *d*_GaN_ = 30 nm and *τ* = 1 × 10^−12^ s)

In Fig. 8, we further examine the effects at different electron drift velocities. In Fig. 8a, we can observe that both the resonant frequency and power increase linearly with the rise in electron drift velocity. This can also be explained by the results in Fig. 8b. As the electron drift velocity increases, the time required for the current density to reach saturation becomes shorter, while the saturation current density gradually rises. At the same time, the radiation spectra at different electron drift velocities are plotted in Fig. 8a (inset). It can be observed that the electron drift velocity primarily influences the radiation power, while its effect on the resonance frequency is not particularly significant. When the electron drift velocity *v*_0_ = 2 × 10^5^ m/s, the maximum calculated radiation power can reach up to 2.5 mW.

We can theoretically estimate the radiation power of N-polar GaN devices. In N-polar GaN devices, the maximum radiation intensity is limited by the gate voltage swing. Since the distance between the gate and the channel is very small, the gate and the channel can be considered as forming a parallel plate capacitor. Hence, the maximum radiation power *P* can be estimated as *C*$U_{0}^{2}$*Wv*_s_*α*^2^ [4]. Assuming *α* ≈ 0.5, *d* ≈ 0.5 nm, *s* = 1.15 × 10^8^ V, *W* = 600 μm, *L*_g_ = 100 nm, and *n*_s_ = 2 × 10^13^ cm^−2^, we obtain *P* = 24 mW at the frequency *f* ≈ *s*/(4*L*_g_) ≈ 5 THz. Compared to the estimated 2 mW output power at 1.5 THz reported in Ref. [4], N-polar GaN devices, due to their higher electron gas concentration, are theoretically capable of generating higher radiation power (24 mW) and achieving a higher operating frequency (2.5 THz).

## Conclusion

In this paper, we analyze and use numerical methods to study the THz radiation characteristics of GaN HEMTs with N-polar surfaces based on DS instability. By simultaneously solving Maxwell’s equations and the self-consistent hydrodynamic model, we demonstrate that under asymmetric boundary conditions, N-polar GaN HEMTs can exhibit plasma wave instability, leading to terahertz radiation. We have considered the effects of GaN channel layer thickness, gate length, channel carrier density, and channel electron drift velocity on plasma wave instability and terahertz radiation. The simulation results based on Dyakonov–Shur instability offer valuable insights for the future design of high-power terahertz on-chip sources utilizing N-polar AlGaN/GaN HEMTs.

## Data Availability

The data set that this work is based upon is available from the corresponding author upon reasonable request. The results in this study were obtained by MATLAB and the code is available from the corresponding author upon reasonable request.
